# Persistence of Virus Reservoirs in ART-Treated SHIV-Infected Rhesus Macaques after Autologous Hematopoietic Stem Cell Transplant

**DOI:** 10.1371/journal.ppat.1004406

**Published:** 2014-09-25

**Authors:** Maud Mavigner, Benjamin Watkins, Benton Lawson, S. Thera Lee, Ann Chahroudi, Leslie Kean, Guido Silvestri

**Affiliations:** 1 Emory Vaccine Center and Yerkes National Primate Research Center, Emory University, Atlanta, Georgia, United States of America; 2 Aflac Cancer and Blood Disorders Center, Children's Healthcare of Atlanta and Emory University School of Medicine, Atlanta, Georgia, United States of America; 3 Center for Immunology and Vaccines, Children's Healthcare of Atlanta and Emory University School of Medicine, Atlanta, Georgia, United States of America; 4 Department of Pediatrics, Emory University School of Medicine, Atlanta, Georgia, United States of America; Vaccine Research Center, United States of America

## Abstract

Despite many advances in AIDS research, a cure for HIV infection remains elusive. Here, we performed autologous hematopoietic stem cell transplantation (HSCT) in three Simian/Human Immunodeficiency Virus (SHIV)-infected, antiretroviral therapy (ART)-treated rhesus macaques (RMs) using HSCs collected prior to infection and compared them to three SHIV-infected, ART-treated, untransplanted control animals to assess the effect of conditioning and autologous HSCT on viral persistence. As expected, ART drastically reduced virus replication, below 100 SHIV-RNA copies per ml of plasma in all animals. After several weeks on ART, experimental RMs received myeloablative total body irradiation (1080 cGy), which resulted in the depletion of 94–99% of circulating CD4+ T-cells, and low to undetectable SHIV-DNA levels in peripheral blood mononuclear cells. Following HSC infusion and successful engraftment, ART was interrupted (40–75 days post-transplant). Despite the observed dramatic reduction of the peripheral blood viral reservoir, rapid rebound of plasma viremia was observed in two out of three transplanted RMs. In the third transplanted animal, plasma SHIV-RNA and SHIV DNA in bulk PBMCs remained undetectable at week two post-ART interruption. No further time-points could be assessed as this animal was euthanized for clinical reasons; however, SHIV-DNA could be detected in this animal at necropsy in sorted circulating CD4+ T-cells, spleen and lymph nodes but not in the gastro-intestinal tract or tonsils. Furthermore, SIV DNA levels post-ART interruption were equivalent in several tissues in transplanted and control animals. While persistence of virus reservoir was observed despite myeloablation and HSCT in the setting of short term ART, this experiment demonstrates that autologous HSCT can be successfully performed in SIV-infected ART-treated RMs offering a new experimental *in vivo* platform to test innovative interventions aimed at curing HIV infection in humans.

## Author Summary

While antiretroviral therapy (ART) can reduce HIV replication, it does not eradicate the virus from an infected individual. Replication-competent viruses persist on ART and our incomplete understanding of these viral reservoirs greatly complicates the generation of a cure for HIV. In this study we performed, for the first time, hematopoietic stem cell transplant (HSCT) in the established model of SIV infection of rhesus macaques (RM). The HSC originating from the bone marrow were collected before SIV infection. After SIV infection, RM were treated with ART for several weeks to reduce viral replication before performing a total body irradiation and a transplant with their own, pre-infection, stem cells. The irradiation eliminated 94–99% of the circulating CD4+ T-cells, the main cell target of HIV/SIV infection. A successful engraftment of the HSC was observed and blood viral reservoirs were drastically reduced. However, when ART was interrupted, a rapid rebound of plasma viremia was observed in two out of three transplanted RM indicating that the massive reset of the hematopoietic compartment was not sufficient to eliminate the total-body virus reservoir in the setting of short term ART. This model of HSCT in SIV-infected RM provides a new platform to investigate HIV eradication strategies.

## Introduction

The introduction of antiretroviral therapy (ART) has dramatically reduced the morbidity and mortality associated with HIV infection and AIDS. However, currently available ART requires life long treatment with significant potential side effects and a cost that places an inordinate burden on public health systems. While reduction of HIV viral loads below detectable limits is often achieved in ART-treated individuals, a treatment that can eradicate or functionally cure HIV infection remains elusive. Many studies indicate that the key obstacle to cure HIV infection is the presence of a persistent reservoir of latently infected cells that are not eliminated by ART [1], [2]. Thus, interruption of ART consistently results in a rebound of viremia to pre-treatment levels [3], [4]. Several biological aspects of this virus reservoir, including its exact cellular and anatomic origin as well as the mechanisms responsible for its establishment and persistence under ART remain poorly understood. This limited knowledge represents a fundamental barrier to a cure for HIV infection, and novel therapeutic strategies aimed at eliminating the reservoir will likely not be developed until we overcome this barrier.

In 2009 it was reported that an HIV-infected individual with acute myelogenous leukemia treated with myeloablative chemotherapy and allogeneic hematopoietic stem cell transplant (HSCT) from a Δ*32ccr5* homozygous donor had remained without detectable HIV replication in the absence of ART for 1.8 years [5], [6]. This first demonstration of a functional cure in this patient was confirmed in 2013 in a follow-up study showing no signs of recrudescent HIV replication and waning of HIV-specific immune responses five years after interruption of ART [7]. More recently, two HIV-infected individuals have been described with prolonged (i.e., 3–8 months) suppression of viremia in absence of ART following allogeneic HSCT from donors homozygous for the wild type *ccr5* allele [8], [9]. Similar to the “Berlin patient” described above, these two transplant recipients were themselves Δ*32ccr5* heterozygotes. The factors involved in the lack of detectable virus replication after ART interruption in HIV-infected individuals undergoing HSCT are complex, and may include (i) the myeloablative regimen involving various combinations of chemotherapy, immunosuppression, and total body irradiation (TBI); (ii) the deficiency of CCR5 in the transplanted donor cells (in the first case); and (iii) a graft versus host effect that may target cells that are latently infected with HIV (i.e., graft versus reservoir effect). Assessing the relative contribution of these factors will likely provide useful information to define the clinical potential of HSCT as a cure for HIV infection.

SIV infection of non-human primates, such as rhesus macaques (RMs) has been used for over two decades as an *in vivo* model for studies of HIV pathogenesis, prevention, and treatment [10]. SIV-infected RMs show remarkable similarities to HIV-infected individuals in terms of mechanisms and markers of disease progression, and current ART regimens can fully suppress virus replication in these animals [11]–[14], thus making this model suitable for probing HIV eradication strategies. In this study, we conducted a controlled test of the contribution of pre-transplant myeloablative irradiation to clearance of the viral reservoir in a cohort of RMs infected with a chimeric simian-human immunodeficiency virus (SHIV) and treated with ART. To the best of our knowledge, this is the first time HSCT has been utilized in RMs to investigate viral persistence. The procedure was successfully performed after SHIV infection and ART-induced control of virus replication using HSCs collected prior to infection. While these recipients showed undetectable plasma viremia and low to absent SHIV-DNA in PBMCs after HSCT, interruption of ART resulted in a rapid rebound of virus replication in two out of three animals. The one transplanted RM who maintained undetectable viremia and SHIV-DNA PCR in PBMCs after ART interruption showed low but detectable levels of SHIV-DNA in sorted circulating CD4+ T-cells, spleen and lymph nodes but not in the gastro-intestinal tract or tonsils. Collectively, these results indicate that the massive reset of the lympho-hematopoietic compartment that follows TBI-induced myeloablation was not sufficient to eliminate the total-body virus reservoir in SHIV-infected RMs in the setting of short term ART. However, this study provides a critical foundation upon which to test other potential contributors to a transplant-mediated cure of HIV.

## Results

### Experimental design

**Figure 1:**
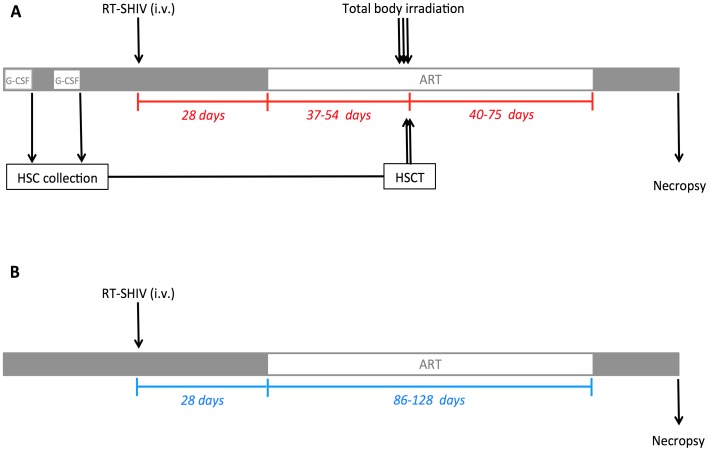
Experimental design. (A) Three RMs received G-CSF 50 mg/kg subcutaneously daily for six consecutive days prior to HSC collection by leukopheresis and cryopreservation of the collected cells. Two apheresis procedures were performed on each transplant recipient. After collection of pre-infection HSCs, RMs were infected i.v. with 10,000 TCID_50_ RT-SHIV_TC_. Starting at day 28 post-infection, RMs received ART daily. After 37 to 54 days on ART, the three experimental RMs underwent TBI (total dose of 1080 cGy), fractionated in three doses given on three consecutive days pre-transplant. On the two following days, the leukopheresis products were infused. ART was interrupted 40 to 75 days post-transplant. (B) Three control RMs were infected i.v. with 10,000 TCID_50_ RT-SHIV_TC_ and received ART for the same period of time as in the transplanted animals. The three control RMs did not undergo TBI/autologous HSCT.

Six RMs were included in this study. All six RMs were males with an average age of 4.2 years (Table 1). Figure 1 shows an overview of the experimental design. Three rhesus macaques (T1, T2, T3) were treated with G-CSF for CD34+ stem cell mobilization followed by HSC collection by leukopheresis and cryopreservation of the collected cells. The six RMs were infected i.v with 10^4^ TCID_50_ RT-SHIV_TC_. Starting at week four post-infection all six RMs were initiated on ART. The ART regimen consisted of two nucleotide/side reverse transcriptase inhibitors (PMPA/tenofovir and FTC/emtricitabine), one non-nucleoside reverse transcriptase inhibitor (efavirenz) and one integrase inhibitor (raltegravir). After five to eight weeks on ART, RMs T1-T3 received myeloablative TBI as pre-transplant conditioning. The leukopheresis products collected before infection were infused within 24 hours following the last dose of TBI. Recipients were given a total of 7.3×10^8^+/−1.3×10^8^ total nucleated cells (TNC)/kg which corresponded to 2.9×10^6^+/−1.1×10^6^ CD34+ cells/kg. After successful engraftment of donor cells (five to eleven weeks post-transplant), ART was interrupted in RMs T1-T3 as well as in the control RMs.

**Table 1:** Animal characteristics and interventions.

			T1	T2	T3	C1	C2	C3	*Mean*
*Animal symbol*			*Filled triangle*	*Filled circle*	*Filled square*	*Open triangle*	*Open circle*	*Open square*	
**Age (years)**			4.3	4.2	4.2	4.2	4.1	4.1	*4.2*
**Gender**			M^a^	M	M	M	M	M	*n/a* b
**Transplant**			+	+	+	−	−	−	*n/a*
**Transplant doses**	**TNC** c **dose/kg**	Infusion 1	1.6×10^8^	2.3×10^8^	3.0×10^8^	n/a	n/a	n/a	*2.3×10^8^*
		Infusion 2	3.0×10^8^	6.6×10^8^	5.3×10^8^	n/a	n/a	n/a	*4.9×10^8^*
		**Total**	**4.6×10^8^**	**8.9×10^8^**	**8.3×10^8^**	n/a	n/a	n/a	***7.3×10^8^***
	**CD34^+^ dose/kg**	Infusion 1	5.3×10^5^	7.3×10^5^	1.2×10^6^	n/a	n/a	n/a	*8.2×10^5^*
		Infusion 2	1.0×10^6^	4.4×10^6^	8.0×10^5^	n/a	n/a	n/a	*2.1×10^6^*
		**Total**	**1.5×10^6^**	**5.1×10^6^**	**2.0×10^6^**	n/a	n/a	n/a	***2.9×10^6^***
**Therapy duration (days)**		Pre-transplant	37	38	54	n/a	n/a	n/a	*42.7*
		Post-transplant	49	40	75^d^	n/a	n/a	n/a	*54.7*
		**Total**	**86**	**78**	**128**	**86**	**78**	**128**	***97.3***

a M: male.

b n/a: non applicable.

c TNC: total nucleated cells.

d PMPA was interrupted at day 36 post-transplant.

### ART control of RT-SHIV replication

**Figure 2:**
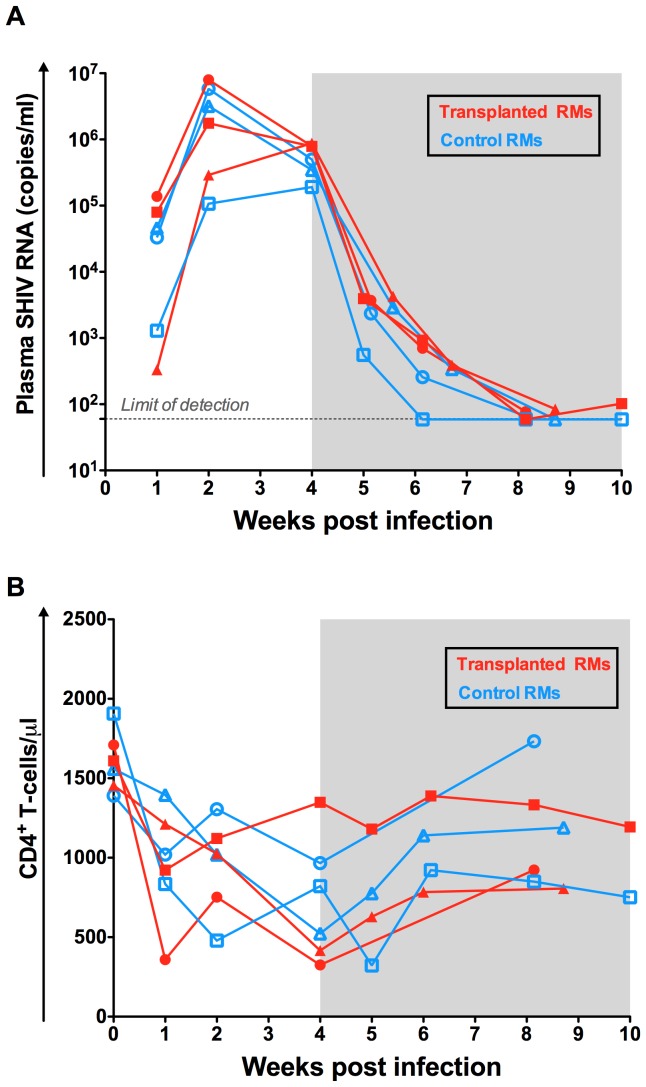
Virologic and immunologic characteristics pre-transplant. (A) The levels of SHIV-RNA, expressed as copies/ml of plasma are shown for each individual animal. Dotted line represents the limit of detection of the assay. (B) Longitudinal assessment of the absolute numbers of circulating CD4+ T-cell expressed as cells per µl. Transplanted animals are depicted in red and controls in blue. Shaded area represents the period of ART treatment.

As shown in Figure 2A, following experimental infection with RT-SHIV_TC_ the six RMs experienced a rapid, exponential increase in virus replication that peaked at week two post infection (10^5^–10^7^ SHIV-RNA copies/ml plasma). ART initiated at week four after infection drastically reduced plasma viral load to less than 100 copies of SHIV-RNA per ml of plasma. Consistent with prior studies of SIV/SHIV infection in RMs, the absolute number of peripheral CD4+ T-cells was decreased following infection and partially restored on ART (Figure 2B).

### Autologous HSCT: Conditioning and engraftment

**Figure 3:**
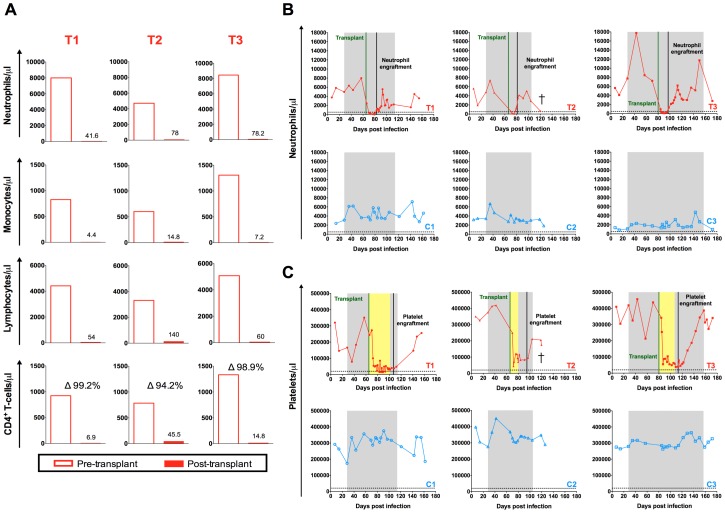
Autologous hematopoietic stem cell transplant. (A) Comparison of the pre- and post-transplant absolute numbers of circulating neutrophils, monocytes, lymphocytes and CD4+ T-cell expressed as cells per µl. Pre-transplant time point is the final assessment prior to TBI (SHIV-infected, on ART). Post-transplant time point represents the nadir cell count observed during the eleven days following TBI. Longitudinal assessment of the absolute numbers of circulating (B) neutrophils and (C) platelets expressed as cells per µl. Transplanted animals are depicted in red, controls in blue. Shaded area represents the period of ART treatment. Yellow area represents the period of platelet transfusion support. The dotted lines indicate the minimum level of neutrophils or platelets used to define engraftment.

The myeloablative TBI resulted in a drastic reduction of the absolute count of blood cells including neutrophils, monocytes, lymphocytes and CD4+ T-cells (Figure 3A). The nadir was observed at day eleven post-TBI for neutrophils (41.6–78.2 neutrophils/µl), day seven post-TBI for monocytes (4.4–14.8 monocytes/µl), and between day one and day five post-TBI for lymphocytes and CD4+ T-cells (54–60 lymphocytes/µl and 6.7–45.5 CD4+ T-cells/µl). Of note, between 94.2 and 99.2% of circulating CD4+ T-cells were eliminated by the TBI (Figure 3A). Engraftment was demonstrated by increasing neutrophil and platelet counts unsupported by transfusion. Neutrophil engraftment was defined as an absolute neutrophil count (ANC) exceeding 500 cells/µl for three consecutive days. The first of these three consecutive days was considered the day of engraftment. As shown in Figure 3B, neutrophil engraftment was successfully achieved between day sixteen and day eighteen post-HSC infusion in the three transplanted animals. During HSCT, the three transplanted animals received platelet and whole blood transfusions for thrombocytopenia prior to platelet engraftment, as well as several antimicrobial prophylactic interventions (Figure S1). Platelet engraftment was defined as a blood platelet count exceeding 20,000 cells/µl in absence of transfusion support for seven consecutive days. According to this definition, platelet recovery was achieved at 42, 22 and 33 days post-transplant for T1, T2 and T3, respectively (Figure 3C).

### Effect of autologous HSCT on CD4+ T-cells

**Figure 4:**
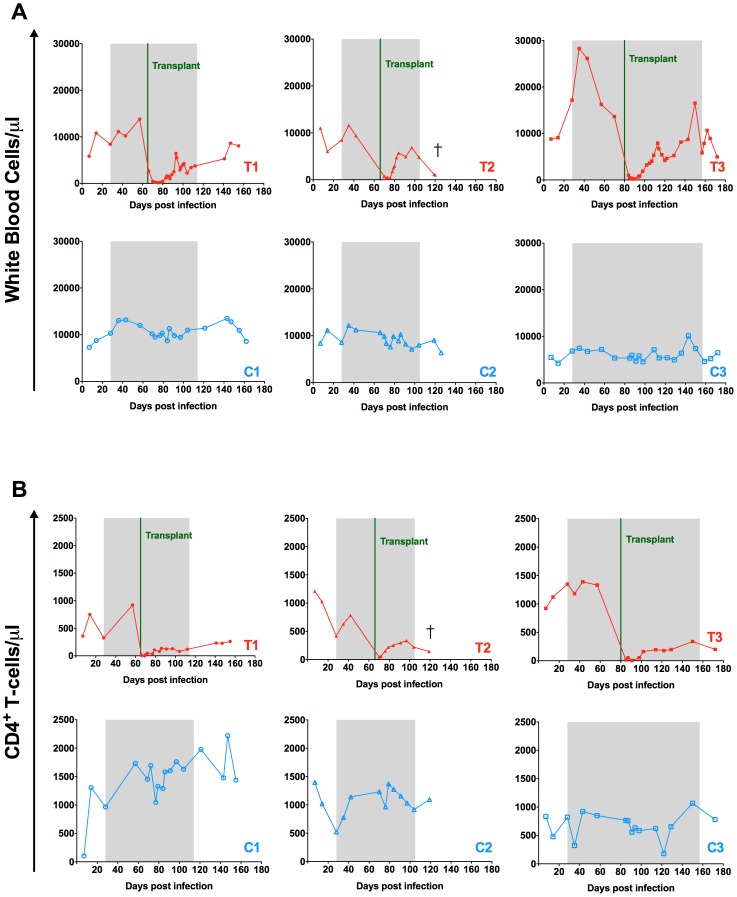
Effect of autologous HSCT on white blood cell and CD4^+^ T-cell counts. Longitudinal assessment of the absolute numbers of white blood cells (A) and circulating CD4^+^ T-cells (B) expressed as cells per µl are shown for each individual animal. Transplanted animals are depicted in red, controls in blue. Shaded area represents the period of ART treatment.

Following transplantation and engraftment, we observed a rapid increase in the absolute leukocyte count and a slower reconstitution of the circulating CD4+ T-cells (Figure 4A and B). The peripheral reconstitution of CD4+ T-cells appeared to involve peripheral T-cell expansion as evidenced by the increased proportion of circulating CD4+ T-cells expressing the proliferation antigen Ki-67 (Figure S2A). In addition, HLA-DR and CCR5 were increased on CD4+ T-cells following HSCT (Figure S2B,C). Further immunophenotypic analyses revealed a significant increase in the proportion of memory CD4+ T-cells (including memory stem cells, central memory, and effector memory) following transplantation (p = 0.03, Figure S3), similar to previous reports of both autologous and allogeneic HSCT [6], [15]. These results are consistent with CD4+ T-cells recovery occurring primarily through the homeostatic proliferation of memory CD4+ T-cells post-transplant.

### Effect of myeloablative irradiation-based pre-transplant conditioning on virus replication and the peripheral blood viral reservoir

**Figure 5:**
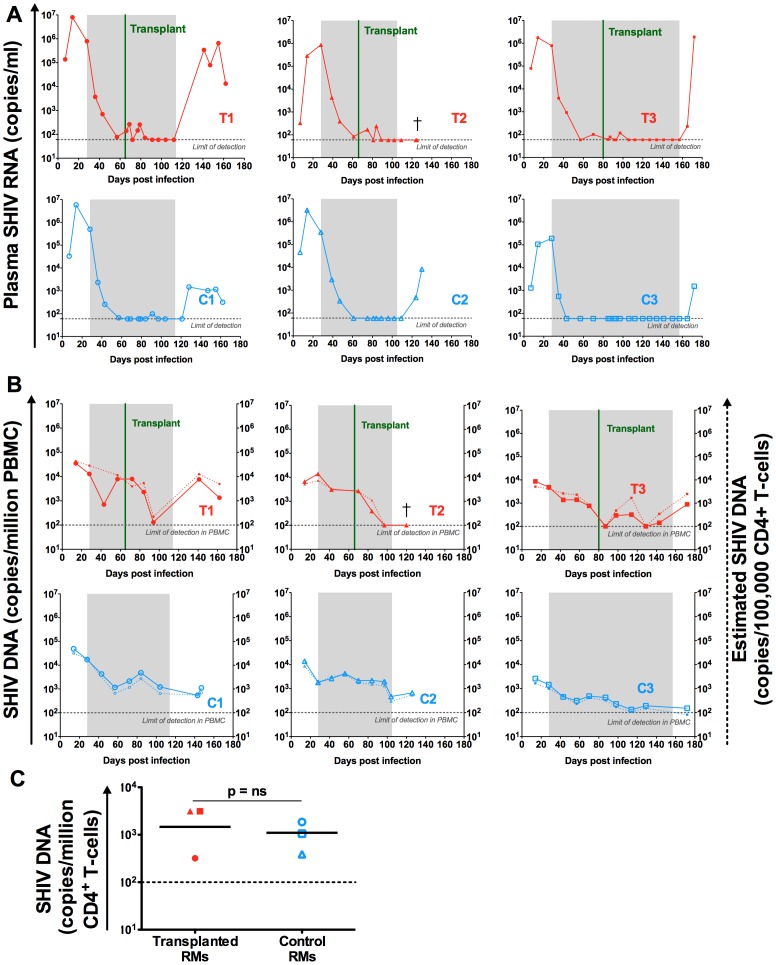
SHIV-RNA and -DNA levels post ART interruption. (A) Longitudinal assessment of the level of SHIV-RNA, expressed as copies/ml of plasma. (B) Longitudinal assessment of cell associated SHIV-DNA. Plain lines represent the level of SHIV-DNA in PBMCs determined by PCR. The dashed lines represent the estimated level of SHIV-DNA in CD4+ T-cells calculated based on PBMC frequency of infection determined by PCR and the frequency of CD4 + T-cells in PBMC determined by flow cytometry. Shaded area represents the period of ART treatment. (C) SHIV-DNA levels determined by PCR at necropsy, in sorted peripheral CD4+ T-cells and expressed as copies/million cells. Lines are drawn at the geometric mean. Mann Whitney U test was used to determine significance. Transplanted animals are depicted in red, controls in blue. Grey dotted lines represent the limit of detection of the assay.

A few blips of transient low-level viremia were observed in the plasma of the three transplanted animals immediately after TBI and HSC infusion and while still on ART (Figure 5A). The origin of these transient increases in viral load is not clear, but it may represent release of virus from pre-existing reservoirs in the setting of events of CD4+ T-cell activation during conditioning and the peri-transplant period. With the exception of these transient episodes of viremia, the plasma viral load remained undetectable in all six animals on ART (Figure 5A). Of note, the ART regimen alone reduced the level of SHIV-DNA in PBMCs (i.e., the peripheral viral reservoir) by 1.0–1.5 log in the three control RMs (Figure 5B). In the transplanted animals, the reduction in cell-associated viral DNA was more pronounced, with two RMs showing levels of SHIV-DNA in PBMCs below the limit of detection and one RM (T1) close to this level (as low as 130 copies/million PBMC, Figure 5B). The normalization of the cell-associated SHIV-DNA level to the CD4+ T-cells counts suggest a decrease in the frequency of infection of these cells post-transplant (Figure 5B).

### Effect of ART interruption on virus replication

ART was interrupted after stem cell engraftment (between 78 and 128 days post-initiation, Table 1). As expected, a rapid viral rebound was observed in the plasma of the three control animals as early as one week post ART cessation (Figure 5A). Two out of the three transplanted animals also experienced a rapid plasma viral rebound post ART interruption. The remaining transplanted animal (T2) maintained an undetectable plasma viral load at two weeks post ART interruption (Figure 5A). Unfortunately, further time-points were not analyzed in this animal as he was euthanized due to progressive renal failure. As shown in Figure 5B, ART interruption led to an increase of the SHIV-DNA levels in the PBMCs of the two transplanted RMs who also experienced a plasma viral rebound. This rebound in PBMC SHIV-DNA was observed at the first assessment post-ART interruption in both animals (day 28 for T1 and day 15 for T3). Of note, no SHIV-DNA was detected in the PBMCs of RM T2 who also maintained undetectable plasma viral load at two weeks after ART interruption. However, further analyses of this animal revealed low but detectable levels of SHIV-DNA in sorted peripheral CD4+ T-cells obtained at the same time-point (i.e., two weeks after ART interruption at necropsy) (Figure 5C).

### SHIV-DNA viral load in tissues after ART interruption

**Figure 6:**
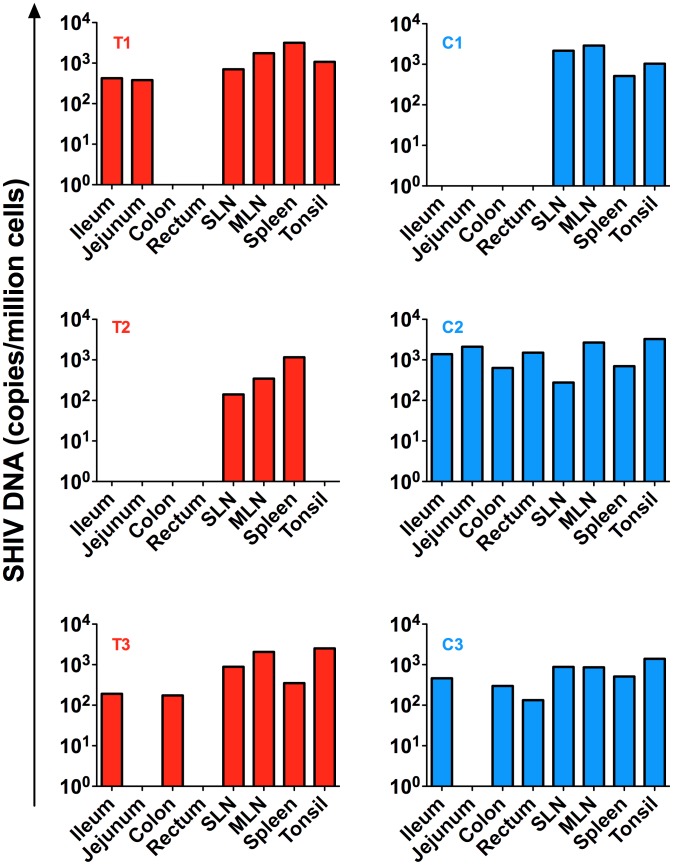
SHIV-DNA in tissues post ART interruption. SHIV-DNA levels expressed as copies/million cells obtained at necropsy from the ileum, jejunum, colon, rectum, superficial and mesenteric lymph nodes, and tonsils are shown for each individual animal. Transplanted animals are depicted in red, controls in blue. Estimated number of CD4+ T-cells per million cells in tissues at necropsy is indicated in Table S1.

Several tissues were collected at necropsy including ileum, jejunum, colon, rectum, superficial and mesenteric lymph nodes as well as tonsils. SHIV-DNA levels in cell suspensions obtained from these tissues were quantified by PCR. As shown in Figure 6, low levels of SHIV-DNA were detected in the spleen and lymph nodes of the transplanted RM who maintained an undetectable peripheral viral load post ART interruption (T2) but not in the tonsils or gut compartments. Of note, we were able to detect SHIV-DNA in the gut and tonsils of the other two transplanted RMs (T1 and T3) who exhibited a rapid rebound of viremia after ART interruption.

## Discussion

The apparent cure of HIV infection in the “Berlin patient” [5]–[7] has energized efforts to understand the mechanisms of virus persistence despite ART-mediated suppression of virus replication. The factors thought to be involved in the favorable outcome of the Berlin patient following HSCT include (i) the myeloablative conditioning regimen; (ii) the donor's homozygosity for Δ*32ccr5*; and (iii) the graft versus host effect. In this test-of-concept study of autologous HSCT in SHIV-infected RMs we interrogated the relative contribution of a myeloablative conditioning regimen in eliminating the persistent reservoir of latently infected cells. To the best of our knowledge this is the first time that a study of similar design has been conducted.

The key findings of this study are the following: (i) autologous HSCT using apheresis products collected prior to infection is feasible in SHIV-infected RMs; (ii) as expected, the myeloablative TBI used for conditioning induced a massive reset of the lympho-hematopoietic compartment, consequently resulting in the depletion of 94.2–99.2% of circulating CD4+ T-cells; (iii) animals receiving autologous HSCT under ART exhibited a prompt and pronounced decline in the peripheral blood viral reservoir (with undetectable SHIV-DNA in PBMCs in two out of three RMs) and maintained undetectable SHIV-RNA viremia with the exception of a few minor blips; (iv) two of the three transplanted RMs showed a very rapid rebound of viremia after ART interruption; and (v) the third transplanted RM, who was sacrificed for clinical reasons at day fourteen post ART interruption, had no detectable virus in plasma, PBMCs, tonsils, and GI tract, low but detectable levels of SHIV-DNA in sorted peripheral CD4+ T-cells and lymph nodes, and moderate levels of SHIV-DNA in the spleen.

Due to many logistical challenges of this experiment we chose to conduct the study in a temporally compressed fashion, with 37–53 days of ART before autologous HSCT, and interruption of ART after hematopoietic reconstitution, rather than prolonged continuation of therapy. This study was therefore designed to determine the impact of myeloablative irradiation on the viral reservoir, rather than the impact of prolonged viral suppression in conjunction with myeloablation. It is therefore possible that a similarly designed study, in which ART is maintained for a significantly longer period both before and after autologous HSCT, would have a different outcome, possibly demonstrating a more dramatic effect of autologous HSCT on the persistent reservoir of latently infected cells. Moreover, we cannot rule our the possibility that the level of virus suppression achieved by the short-term ART regimen in this experiment might not be as complete as what is observed in HIV-infected individuals on long-term ART. In this model of SHIV-infected RM, 5 to 7 weeks on ART pre-transplant may have been insufficient to fully suppress viral replication and the transient low-level viremia observed immediately post-transplant could be attributed to an insufficient period of ART pre-transplant. However, similar viral blips were observed in one patient who received allogeneic stem cell transplant after many years on combined ART [8]. Although the origin of these transient blips is unknown, it may represent release of the virus from latently infected cells in the setting of cell activation during conditioning and the peri-transplant period. In keeping with this hypothesis, it should be noted that in our study the post-transplant period was characterized by an expansion of CD4+ T-cells expressing CCR5 as well as proliferation and activation markers. Together with the observed increased proportion of memory CD4+ T-cells post-transplant, these results suggest that the CD4+ T-cell compartment recovered primarily through homeostatic proliferation of memory CD4+ T-cells.

The myeloablative TBI used for conditioning resulted in the depletion of 94.2–99.2% of circulating CD4+ T-cells. Unfortunately, due to the clinical challenges of this innovative experiment, no tissue biopsies could be obtained immediately post-transplant to evaluate the TBI-induced CD4+ T-cell depletion in tissues. However, this study shows that myeloablative TBI and autologous HSCT did not prevent a rebound of viremia post-ART interruption in two out of three RMs despite relatively early ART initiation (day 28 post-infection). Moreover, while the SHIV-DNA level in PBMCs was undetectable or close to undetectable post autologous HSCT, it rapidly rebounded after ART interruption to levels that were similar or higher than those observed in the control animals at the same time-point. While in the third animal (T2) there was no sign of virus present in the plasma, PBMCs, and various tissues at the time of necropsy, this RM had to be sacrificed due to kidney failure at day fourteen after ART interruption making the interpretation of these data somewhat difficult. Of note, this study was not designed to identify the cellular and anatomic sources of the rapid plasma viral rebound observed in two transplanted RMs following ART interruption. Determining the relative contribution of tissue CD4+ T-cells, macrophages, and potentially other sources represents an important area for future investigation, amenable for interrogation with this model.

We acknowledge a number of limitations in our study including the small number of animals and the foreshortened time line involved. However, the demonstrated feasibility of this test-of-concept study in a non-human primate model of AIDS virus infection is per se an important result given the extreme complexity of the experimental protocol. The RMs included in this study underwent a series of procedures that have been only rarely, if ever, used in the same animal, including stem cell mobilization and harvesting by apheresis, RT-SHIV infection, daily four-drug ART administration, total body irradiation, re-infusion of HSCs, repeated platelet transfusions, and receipt of several antimicrobial prophylaxes. The feasibility of HSCT in SIV- or SHIV-infected RMs suggests, in our view, that further studies using this model in conjunction with longer term ART as well as additional interventions aimed at purging both the peripheral blood and lymphoid tissue-based viral reservoirs will provide critical information for the requirements to cure HIV infection in humans.

With respect to our understanding of the mechanisms responsible for “curing” HIV infection in the Berlin patient, our study supports the hypothesis that myeloablative TBI can cause a significant decrease in the viral reservoir in circulating PBMCs, even though it was not sufficient to eliminate all reservoirs. While the conditioning regimen in the Berlin patient also included antithymocyte globulin and chemotherapy, the use of a Δ*32ccr5* homozygous donor and/or the presence of graft versus host disease likely played a significant role in that clinical context. The importance of graft versus host disease that effectively results in a “graft versus reservoir” effect is also emphasized by the recent observation of two HIV-infected patients in which a prolonged (i.e., 3–8 months) period of undetectable viremia in absence of ART was observed after allogeneic HSCT from donors with wild-type *ccr5* alleles [9], although these patients did eventually develop rebound of viremia [16]. Future studies of allogeneic HSCT in SIV- or SHIV-infected RMs in the presence or absence of gene therapy interventions to knock out *ccr5* would be very informative in this regard, and may elucidate the mechanism of the sustained cure seen in the Berlin patient but not the above mentioned recipients of donor cells wild type for *ccr5*.

In conclusion, we have conducted the first test-of-concept study of myeloablative irradiation and autologous HSCT in ART-treated SHIV-infected RMs. This experiment demonstrated that autologous HSCT is a feasible intervention that can lead to a marked reduction of the virus reservoir in the peripheral blood, and can be used as an experimental *in vivo* platform to test innovative interventions aimed at curing HIV infection in humans.

## Materials and Methods

### Ethics statement

This study was conducted in strict accordance with USDA regulations and the recommendations in the Guide for the Care and Use of Laboratory Animals of the National Institutes of Health, and were approved by the Emory University Institutional Animal Care and Use Committee (Protocol # YER-20000373-061714). SIV-infected animals were housed in standard non-human primate cages, received standard primate feed as well as fresh fruit and enrichment daily, and had continual access to water. Cages also contained additional sources of animal enrichment including objects such as perching and other manipulanda. Animal welfare was monitored daily. Appropriate procedures were performed to ensure that potential distress, pain, or discomfort was alleviated. The sedatives Ketamine (10 mg/kg) or Telazol (4 mg/kg) were used for blood draws and biopsies. Euthanasia of RMs, using Pentobarbital (100 mg/kg) under anesthesia, was performed only when deemed clinically necessary by veterinary medical staff and according to IACUC endpoint guidelines.

### Animals

Six Indian RMs (*Macaca mulatta*), with exclusion of Mamu B*08 and B*17 positive animals, were included in this study. All animals were housed at the Yerkes National Primate Research Center (Atlanta, GA) and treated in accordance with Emory University and Yerkes National Primate Research Center Institutional Animal Care and Use Committee regulations.

### HSCT protocol

Autologous HSCs were harvested at two separate time points in each animal using our previously described apheresis procedure [17]. Animals were prepared for leukopheresis with epoeitin alfa (nine doses of 150 mg/kg, Amgen), given in the two months prior to leukopheresis to increase red cell mass and thus increase the safety of the leukopheresis procedure and filgastrim (G-CSF, 50 mg/kg intramuscularly daily to a maximum of 300 mg, Amgen) for six days prior to leukopheresis to mobilize HSCs as previously described [18]. The leukopheresis was analyzed for cell content and then cryopreserved in 10% DMSO using standard clinical techniques. Both apheresis units were infused into the transplant recipient within 24 hours of the completion of TBI.

### Analysis of the hematopoietic stem cell product

The leukopheresis products were analyzed by flow cytometry prior to cryopreservation for the total nucleated cell dose, the CD34+ cell dose, CD3+ T-cell dose, CD4+ T-cell dose, CD8+ T-cell dose, and the CD20+ B-cell dose using the following antibodies; CD3 (clone SP34-2), CD34 (clone 563), CD45 (clone D058-1283), CD8 (clone RPA-T8) from BD Biosciences; CD20 (clone 2h7), CD4 (clone OKT4) from eBioscience.

### SHIV infection

The RMs were intravenously (i.v.) infected with 10,000 50% tissue culture infective doses (TCID_50_) of RT-SHIV_TC_. The virus stock was provided by Dr. Tom North (Emory University) and prepared as previously described [19], [20]. The RT-SHIV used for this study had the T-to-C substitution at position 8 of the SIV tRNA primer binding site which is necessary for high replication of RT-SHIV *in vivo* [21].

### Preparation and administration of antiretroviral drugs

Efavirenz was provided by Bristol-Myers Squib, raltegravir was provided by Merck, and emtricitabine (FTC) and tenofovir (PMPA) were provided by Gilead Sciences. Efavirenz was fed at 200 mg per day by mixing the contents of a 200 mg capsule into food. Raltegravir was fed at 100 mg twice daily by mixing the drug into food. Stock solutions of FTC were prepared in phosphate-buffered saline (PBS, pH 7.4). PMPA was suspended in distilled water, with NaOH added to a final pH of 7.0. FTC and PMPA stocks were filter sterilized and stored at 4°C. These drugs were administered subcutaneously, at a dose of 30 mg/kg of body weight once daily. Drug dosages were adjusted weekly according to body weight.

### Pre-transplant preparation

The pre-transplant preparative regimen consisted of myeloablative TBI to a total dose of 10.8 Gy, given in three divided fractions of 3.6 Gy each (at a rate of 7.5 cGy/minute) using a Varian Clinac 23 EX (Varian). Irradiation took place on days −2, −1, and 0 (the day of transplant), with the final dose of irradiation given just prior to infusion of the first of two leukopheresis products.

### Peri-transplant supportive care

Animals were treated with the following empiric antimicrobial agents in the peri-transplant period, as previously described [17], [22]. (Figure S1): (1) Polymixin B (1,000,000 units orally daily, Ben Venue Laboratories, Inc) and neomycin sulfate (500 mg orally daily, Teva Pharmaceuticals). Dosing of both agents was begun on day −7 and continued until neutrophil engraftment (Absolute neutrophil count >500 cells/µl for three consecutive days). (2) Enrofloxacin (7 mg/kg intramuscularly daily, Bayer Healthcare) starting on day −1 and continuing until neutrophil engraftment. (3) Fluconazole (5 mg/kg orally daily, Pfizer) starting on day −1 and continuing until neutrophil engraftment. (4) Cidofovir (5 mg/kg i.v., Gilead) starting on day +6 and continuing once weekly as clinically tolerated, to prevent CMV reactivation. Cidofovir was given to transplant recipients 1 and 2. However, because we observed significant increases in serum creatinine in these recipients, the third transplant recipient was treated with oral valganciclovir (60 mg twice daily, Genentech), which was begun after neutrophil engraftment was observed.

### Transfusional support

Transplanted animals received both platelet rich plasma and whole blood (irradiated at 2200 rad prior to transfusion) to treat thrombocytopenia (platelet count <50×10^6^/ml) or anemia (hemoglobin <10 g/dl) or with the development of clinically significant bleeding. Blood product support adhered to ABO antigen matching principles.

### Sample collections and processing

EDTA-anticoagulated blood samples were collected regularly and used for a complete blood count, routine chemical analysis and immunostaining, with plasma separated by centrifugation within 1 h of phlebotomy. PBMCs were prepared by density gradient centrifugation. CD4+ T-cells were negatively selected from frozen PBMCs using magnetically labeled microbeads and subsequent column purification according to the manufacturer's protocol (Miltenyi Biotec). Tissue samples including ileum, jejunum, colon, tonsils and mesenteric and superficial lymph nodes were collected post-mortem. After two washes in RPMI and removal of connective and fat tissues, gut tissues were cut in small pieces and lymph nodes and tonsils were grinded using a 70-µm cell strainer. Gut cells were isolated by digestion with collagenase and DNase I for 2 h at 37°C and then passed through a 70-µm cell strainer. The cell suspensions obtained were washed and immediately used for immunostaining, cryopreserved or lysed in RLT^+^ buffer and stored at −80°C until use.

### Plasma RNA and cell-associated DNA viral quantification

Plasma viral quantification was performed as described previously [23]. DNA was extracted from PBMCs, sorted peripheral CD4+ T-cells, and tissue cell suspensions using the Blood DNA Mini Kit (QIAGEN). Quantification of SIVmac *gag* DNA was performed as previously described on the extracted cell-associated DNA by quantitative PCR using the 5′ nuclease (TaqMan) assay with an ABI7500 system (PerkinElmer Life Sciences). The sequence of the forward primer for SIVmac *gag* was 5′-GCAGAGGAGGAAATTACCCAGTAC-3′; the reverse primer sequence was 5′-CAATTTTACCCAGGCATTTAATGTT-3′; and the probe sequence was 5′-6 FAM-TGTCCACCTGCCATTAAGCCCGA-TAMRA-3′. For cell number quantification, quantitative PCR was performed simultaneously for monkey albumin gene copy number. All PCR were performed in duplicate with 10,000 cell equivalent per reaction with a limit of detection of 1 copy per reaction.

### Immunophenotype by flow cytometry

Multicolor flow cytometric analysis was performed on whole blood or frozen PBMCs using predetermined optimal concentrations of the following fluorescently conjugated mAbs: CD3-PacBlue or -APC-Cy7 (clone SP34-2), CD95-PE-Cy5 (clone DX2), Ki-67-AF700 (clone B56), HLA-DR-PerCP-Cy5.5 (clone G46-6), CCR7-PE-Cy7 (clone 3D12), CCR5-PE or -APC (clone 3A9), CD45RA-FITC (clone L48), Biotin-CD122 (clone Mik-β3) from BD Biosciences; CD8-BV711 (clone RPA-T8), CD4-APC-Cy7 or -BV650 (clone OKT4), Streptavidin-PE from Biolegend, and CD28-ECD (clone CD28-2) from Beckman-Coulter. Flow cytometric acquisition and analysis of samples was performed on at least 100,000 events on an LSRII flow cytometer driven by the FACSDiva software package (BD Biosciences). Analyses of the acquired data were performed using FlowJo Version 10.0.4 software (TreeStar).

### Statistical analyses

For the comparison of SHIV-DNA in sorted CD4+ T-cells in transplanted and control RMs the nonparametric Mann-Whitney U test was used. For the comparison of the proportion of memory CD4+ T-cells before and after transplant, a Wilcoxon matched-pairs signed rank test was used. Statistical significance was set at p<0.5. All analyses were performed using GraphPad Prism v4.0.

## Supporting Information

Figure S1 **Peri-transplant supportive care.**
(PDF)

Figure S2 **CD4+ T-cell proliferation post-transplant.** Flow cytometric longitudinal assessment of the percentage of circulating CD4+ T-cells expressing the proliferation antigen Ki-67 (A), the activation marker HLA-DR (B) and the HIV/SIV coreceptor CCR5 (C). Transplanted animals are depicted in red, controls in blue. Shaded area represents the period of ART treatment.
(PDF)

Figure S3 **Changes in memory CD4+ T-cell subpopulations following transplant.** (**A**) Comparison of the pre- and post-transplant proportion of circulating naïve and memory CD4+ T-cell subpopulations was performed by flow cytometry using the following markers: naïve (CD28+CD95-CCR7+), memory stem cells (SCM, CD45RA+CCR7+CD28+CD95+CD122+), central memory (CM, CD28+CD95+CCR7+), and effector memory (EM, CD28+/−CD95+CCR7−). Labels indicate percentage of total CD4+ T-cells. CD4+ T-cell subpopulations were analyzed seven days pre-transplant and fourteen to nineteen days post-transplant. (B) Wilcoxon matched-pairs signed rank test was used to compare the proportion of total CD4+ memory T-cells (SCM, central memory and effector memory) in the peripheral blood before and after transplant.
(PDF)

Table S1 **Estimation of the number of CD4+ T-cells per million cells in different tissues at necropsy.**
(DOCX)
