# Supplementary figures and images for: Persistence of Virus Reservoirs in ART-Treated SHIV-Infected Rhesus Macaques after Autologous Hematopoietic Stem Cell Transplant
Source: PLoS Pathog. 2014 Sep 25;10(9):e1004406. doi: 10.1371/journal.ppat.1004406 (PMC4177994; doi:10.1371/journal.ppat.1004406)

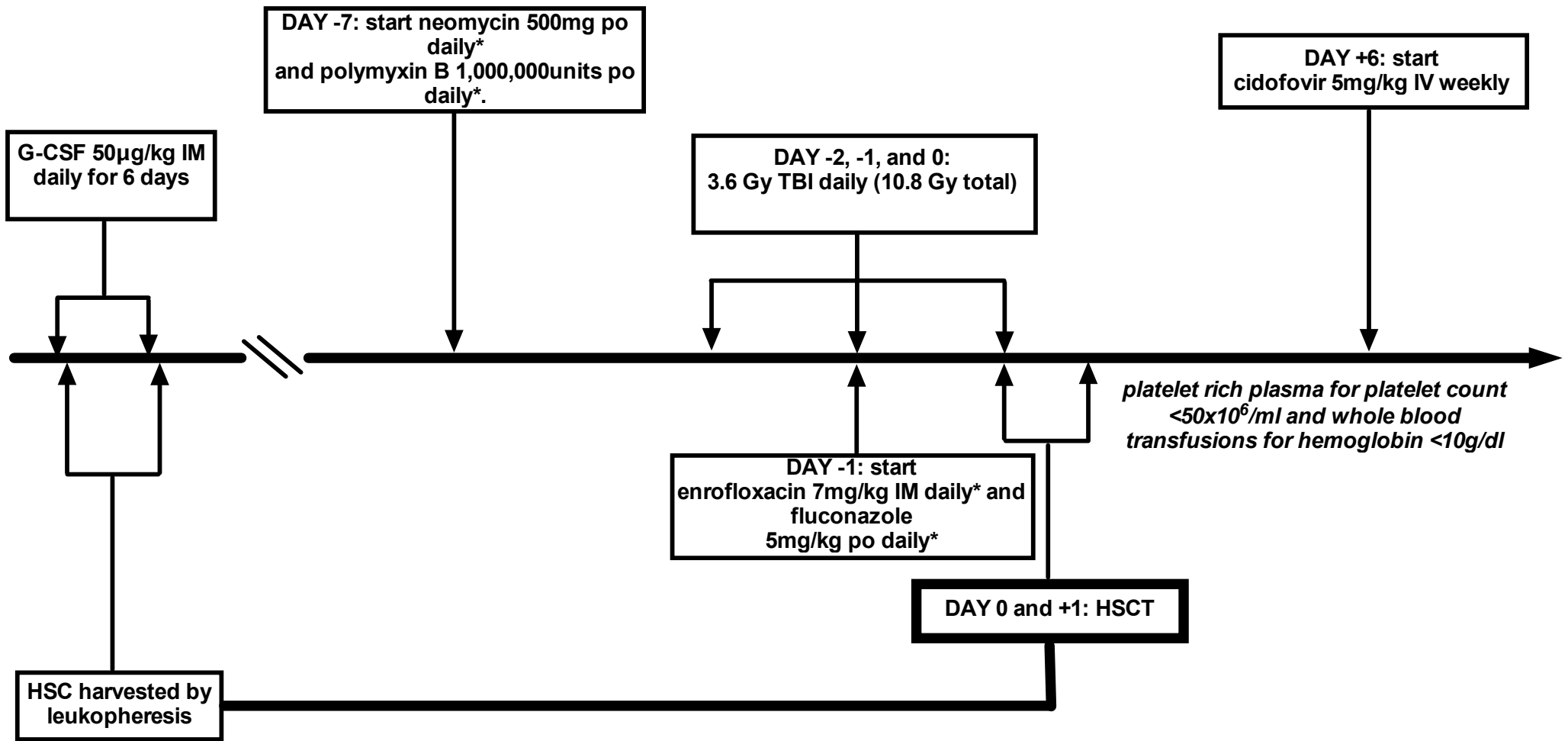

\* Continued until neutrophil engraftment

Supplement: Figure S1 — Peri-transplant supportive care. (PDF) [file ppat.1004406.s001.pdf]

**A****CD4<sup>+</sup> T-cells Ki-67<sup>+</sup> (%)**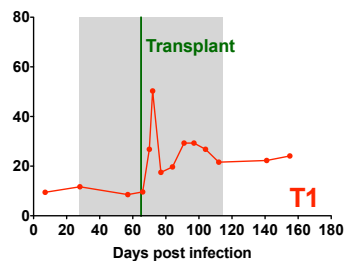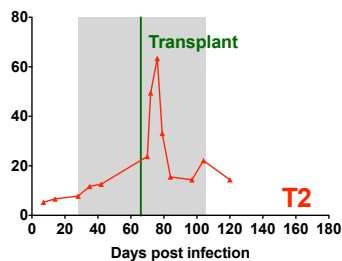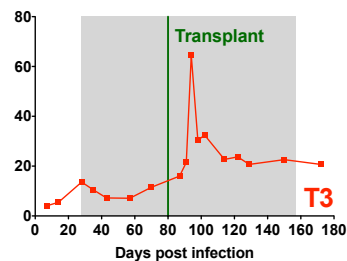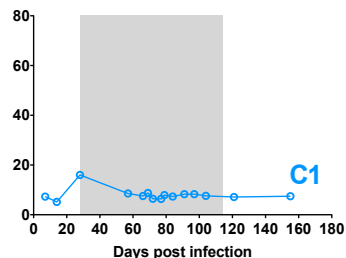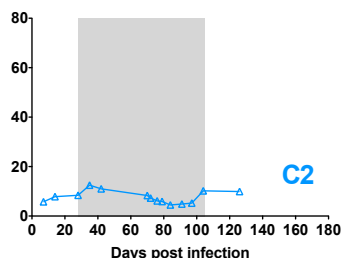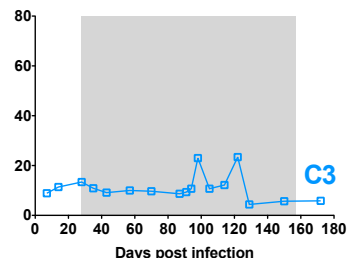**B****CD4<sup>+</sup> T-cells HLA-DR<sup>+</sup> (%)**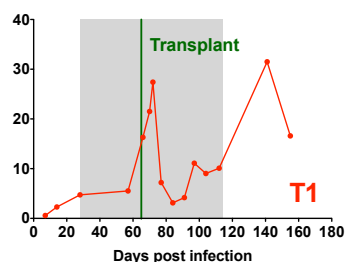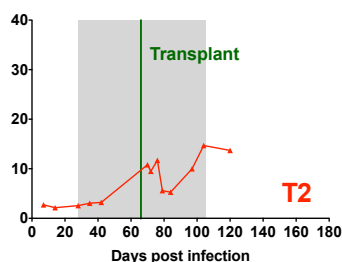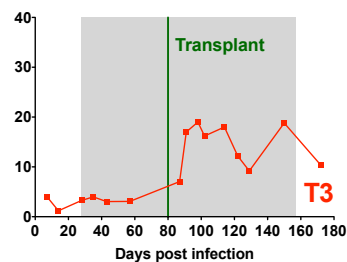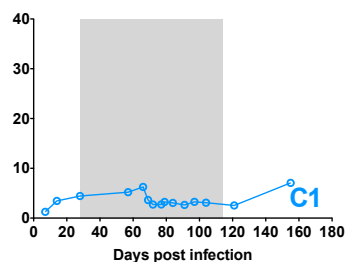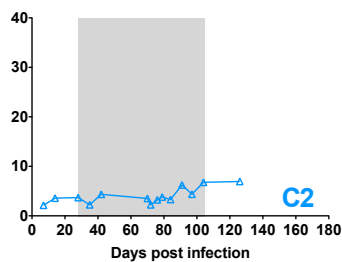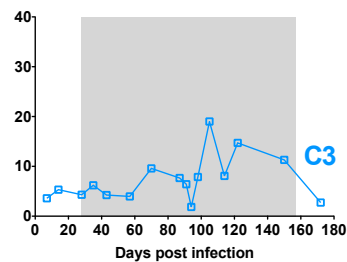**C****CD4<sup>+</sup> T-cells CCR5<sup>+</sup> (%)**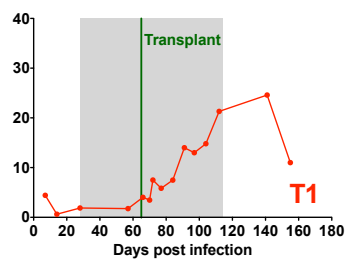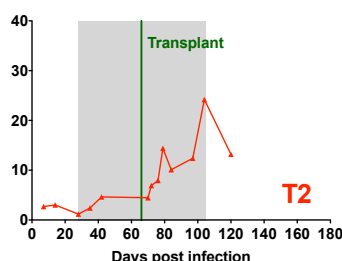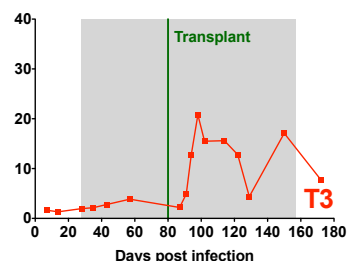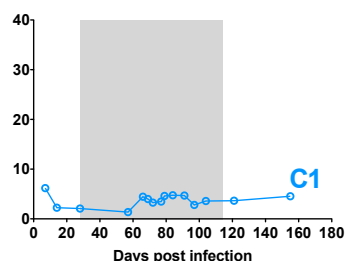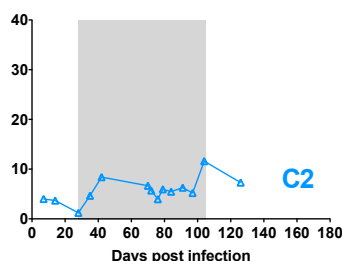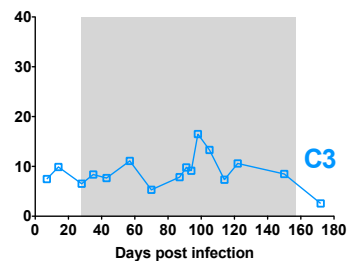

Supplement: Figure S2 — CD4+ T-cell proliferation post-transplant. Flow cytometric longitudinal assessment of the percentage of circulating CD4+ T-cells expressing the proliferation antigen Ki-67 (A), the activation marker HLA-DR (B) and the HIV/SIV coreceptor CCR5 (C). Transplanted animals are depicted in red, controls in blue. Shaded area represents the period of ART treatment. (PDF) [file ppat.1004406.s002.pdf]

A

Pre-transplant

Post-transplant

T1

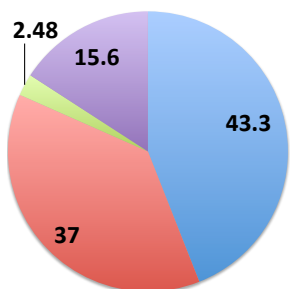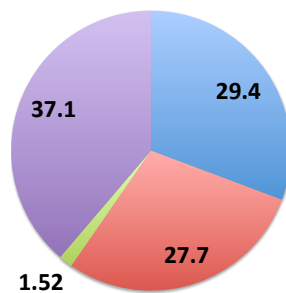

T2

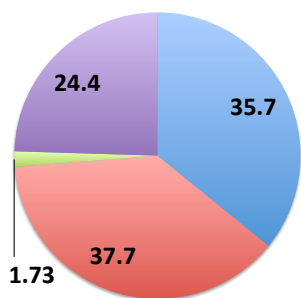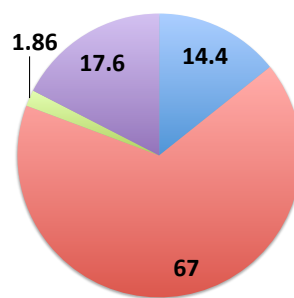

T3

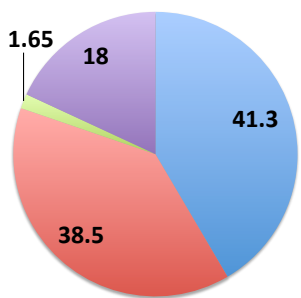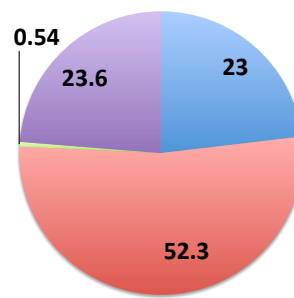

B

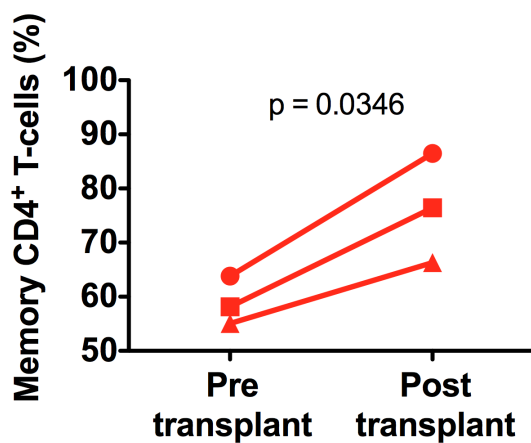

Supplement: Figure S3 — Changes in memory CD4+ T-cell subpopulations following transplant. (A) Comparison of the pre- and post-transplant proportion of circulating naïve and memory CD4+ T-cell subpopulations was performed by flow cytometry using the following markers: naïve (CD28+CD95-CCR7+), memory stem cells (SCM, CD45RA+CCR7+CD28+CD95+CD122+), central memory (CM, CD28+CD95+CCR7+), and effector memory (EM, CD28+/−CD95+CCR7−). Labels indicate percentage of total CD4+ T-cells. CD4+ T-cell subpopulations were analyzed seven days pre-transplant and fourteen to nineteen days post-transplant. (B) Wilcoxon matched-pairs signed rank test was used to compare the proportion of total CD4+ memory T-cells (SCM, central memory and effector memory) in the peripheral blood before and after transplant. (PDF) [file ppat.1004406.s003.pdf]
