# Supplementary material for: Persistence of Virus Reservoirs in ART-Treated SHIV-Infected Rhesus Macaques after Autologous Hematopoietic Stem Cell Transplant
Source: PLoS Pathog. 2014 Sep 25;10(9):e1004406. doi: 10.1371/journal.ppat.1004406 (PMC4177994; doi:10.1371/journal.ppat.1004406)
Supplement: Table S1 — Estimation of the number of CD4+ T-cells per million cells in different tissues at necropsy. (DOCX) [file ppat.1004406.s004.docx]

**Table S1. Estimation of the number of CD4+ T-cells per million cells in different tissues at necropsy.**

|  | **T1** | **C1** | **T2** | **C2** | **T3** | **C3** |
| --- | --- | --- | --- | --- | --- | --- |
| Ileum | 2,300 | 4,500 | 11,500 | 50,700 | 1,600 | 21,800 |
| Jejunum | 2,000 | 12,800 | 93,400 | 19,400 | 2,900 | 5,500 |
| Colon | 200 | 2,200 | 53,900 | 53,200 | 810 | 7,900 |
| Rectum | 400 | 3,100 | 29,100 | 34,200 | 90 | 3,000 |
| SLN | 41,300 | 373,000 | 517,000 | 398,000 | 106,000 | 313,000 |
| MLN | 67,800 | 438,000 | 528,000 | 417,000 | 74,500 | 299,000 |
| Spleen | 18,700 | 138,000 | 111,000 | 142,000 | 30,300 | 130,000 |
| Tonsils | 6,900 | 171,000 | 173,000 | 147,000 | 107,000 | 227,000 |
